# The Influence of Mixing Methods on the Compressive Strength and Fluoride Release of Conventional and Resin-Modified Glass Ionomer Cements

**DOI:** 10.1155/2019/6834931

**Published:** 2019-09-15

**Authors:** Gilliard Lima Oliveira, Ceci Nunes Carvalho, Edilausson Moreno Carvalho, José Bauer, Adriana Mara Araújo Leal

**Affiliations:** ^1^Department of Restorative Dentistry, School of Dentistry, University Ceuma (UNICEUMA), R. Josué Montello, 1, Renascença II, Zip Code 65075120, São Luis, Maranhão, Brazil; ^2^Discipline of Dental Materials, School of Dentistry, Federal University of Maranhão (UFMA), Av. dos Portugueses, 1966, Zip Code 65085680, São Luis, Maranhão, Brazil

## Abstract

**Objective:**

To evaluate the compressive strength and fluoride ion release of conventional and resin-modified glass ionomer cement mixing methods (hand mix and mechanical mix) compared to ready-to-use ones.

**Materials and Methods:**

Two conventional glass ionomer cements (GICs) (Fuji II and Fuji II Caps), two resin-modified GICs (Fuji II LC and Fuji II L Caps), and one ready-to-use GIC (Ionoseal, Voco) were used. For the compressive strength test, cylindrical specimens (6 mm × 4 mm) of each group were prepared. The test was performed in a universal testing machine (EMIC DL2000). For the fluoride release test, specimens were prepared in the form of discs and placed in deionized/distilled water, which were replaced daily for 15 days. The fluoride ion release readings were performed on an electrode (Orion 96-09) connected to a digital ion analyzer (Quimis 0400ISE). The compressive strength data were analyzed with one-way ANOVA, and the ion release data were submitted to repeated measures ANOVA (material vs. time) and Holm–Sidak post test (*α* = 5%).

**Results:**

The one-way ANOVA showed statistical difference between the tested materials (*p* < 0.001). Ionoseal showed the highest values of compressive strength (*p* < 0.001). Mechanical manipulation increased the compressive strength only for conventional GIC, and resin-modified GIC did not present any statistical difference. Conventional GIC (mechanical mix) showed higher fluoride release on first day than the other groups tested.

**Conclusion:**

There was influence of the mixing methods of the materials on the compressive strength and fluoride release pattern of the glass ionomer cements.

## 1. Introduction

The clinical application of glass ionomer cement (GIC) as a restorative material has been indicated in dentistry for more than 40 years [1]. Over time, some changes have been made in the composition of these materials with the aim to improve its mechanical, aesthetic, and biological properties [2–4]. At the end of the 1980's, monomeric components such as bisphenol-A-glycidyl methacrylate (Bis-GMA), urethane dimethacrylate (UDMA), and 2-hydroxyethyl methacrylate (HEMA) were added to the ionomer composition, resulting in resin-modified GICs [4–7]. This innovation was motivated by an attempt to overcome the problems associated with conventional ionomer cements, such as low cohesive strength, low wear resistance, and handling-related sensitivity [8–11].

Historically, glass ionomers require hand mixing, which can be a time-consuming step in the clinic [12]. Beyond the time consumption, manual manipulation may suffer from variations caused by the operator, due to imprecise dispensing of the powder and liquid, since the volume of the powder depends on the density of the powder in the scoop and the volume of the liquid depends on the angulation of the bottle at the time of drop dispensation [13]. In clinical practice, these variations are exacerbated when the operator does not carefully follow the manufacturer's recommendations, which results in improper manipulation and interferes in the physical and mechanical properties of the material [14].

The difficulty of obtaining a correct proportioning of the powder and liquid, with subsequent proper manual manipulation, has led to a trend of commercialization of GICs into prefabricated capsules. These materials, after disruption of the membrane separating the powder of the liquid, must be mechanically manipulated in a mixer, presenting defined proportions and time [15]. The use of encapsulated materials facilitates the agglutination and insertion in the cavity. This can reduce the inclusion of porosities inside the material, which contributes to maintain advantageous characteristics and minimize their disadvantages [14, 16, 17]. However, this demands a higher cost due to the need for equipment for mixing.

More recently, light curing glass ionomer composite cements ready to use (no mixing), with fast light cure (20 seconds) and high compressive strength and fluoride release became available in the market. A ready-to-use material provides faster patient care as well as no need for manipulation tools such as plates, spatulas, and equipment used for encapsulated ionomer, which reduces costs. In addition, ready-to-use cement can be free of bubbles, as it does not require manipulation.

Due to the large-scale use of different ionomer cements, the present study investigated the effect of mixing methods on the compressive strength and fluoride release of different GICs. The null hypothesis was as follows: the mixing methods (hand mixing, mechanical mixing, and read-to-use) of the cements have no effects on (i) its compressive strength and (ii) fluoride release.

## 2. Materials and Methods

Five glass ionomer cements were used: two conventional (Fuji II and Fuji II Capsule, GC Corp., Tokyo, Japan), two resin-modified (Fuji II LC and Fuji II LC Capsule, GC Corp., Tokyo, Japan), and one ready for use (Ionoseal, Voco, Germany). The composition of the tested materials is shown in Table 1.

### 2.1. Compressive Strength Test

To evaluate the compressive strength, 36 cylindrical specimens (*n* = 6) were made in a bipartite polytetrafluoroethylene matrix, 6 mm high and 4 mm in diameter, according to ISO 9917-1 and 9917-2 [18, 19].

For the ionomer presented in the powder/liquid system, the manipulation was performed according to the manufacturer's recommendations. The encapsulated ionomers were mechanically manipulated in an amalgamator (Ultramat S, SDI, Victoria, Australia) for the period recommended by the manufacturer. To minimize the incorporation of air bubbles into the specimens, manually manipulated material was inserted into the matrix with the aid of a Centrix syringe (DFL, Rio de Janeiro, RJ Brazil), and the encapsulated material was inserted with the aid of a metal insertion syringe (SDI, Victoria, Australia). Then, a polyester strip was placed on top of the matrix and a slight pressure was applied with a glass slab to remove the excess material.

Specimens of the conventional ionomer were held in the matrix for 1 h and subsequently removed for visual inspection. Those who presented defects were discarded. Resin-modified GIC specimens were photoactivated following the manufacturer's recommendations using a light curing unit (Optilux 501, Kerr, Orange, CA, USA). After 1 h, the specimens were removed and those that contained defects were discarded. The excesses were removed through hand lapping on silicon carbide (#600) paper using water. The specimens were stored after removal of the matrix for 23 hours, with a total storage time of 24 hours. All selected samples were stored in distilled water at 37°C for 24 hours. The compressive strength of the specimens was determined using a universal testing machine (EMIC DL2000, São José dos Pinhais, PR, Brazil) with a crosshead speed of 0.5 mm/min.

### 2.2. Fluoride Release Analysis

Four specimens (4.15 mm diameter × 2.30 mm height) of each material were made into a disposable cylindrical polytetrafluoroethylene matrix with disc format. All restorative materials were prepared the same way as that of the compressive strength test. Each specimen was placed in a polyethylene vial filled with 2 mL of deionized water, which was changed daily for 15 days' period.

The amount of fluoride (ppm) was measured using an ion electrode (Quimis, Model Q400ISE, Diadema, SP, Brazil) coupled to a digital pH/F∼analyzer (Quimis, Model Q838-F-Diadema, SP, Brazil), previously calibrated with a series of standard solutions at different concentrations of fluoride: 1.0, 2.0, 4.0, 8.0, and 16 ppm. Ion release was determined each day after buffering the solutions with equal volumes of TISAB II (Total Ionic Strength Adjustment Buffer, Orion, MA, USA). The solutions were read in triplicate. After each reading, the electrode was washed with deionized water and dried. The readings were performed daily for a period of 15 days. By measuring fluoride in ppm in a known volume of water, it was possible to calculate the total amount of fluoride ions released from the specimens. After each reading, the total fluoride released in micrograms was calculated by multiplying the parts per million (1 ppm = 1 *μ*g/mL) by the water sample volume (2 mL). The total fluoride was then divided by the area of the sample to obtain the fluoride release in micrograms per square centimeter [20].

### 2.3. Statistical Analysis

Statistical analysis was performed using the SigmaPlot 13 software (SigmaPlot v. 13.1, Systat Software Inc., San Jose, USA). The normality and equality of variance assumptions were statistically analyzed by Shapiro–Wilk and Brown–Forsythe tests. The compressive strength means were analyzed by one-way ANOVA and Holm–Sidak tests for comparisons (*α* = 0.05). The fluoride release data were submitted to two-way repeated measures ANOVA (materials vs. time) and Holm–Sidak tests (*α* = 0.05).

## 3. Results

**Figure 1:**
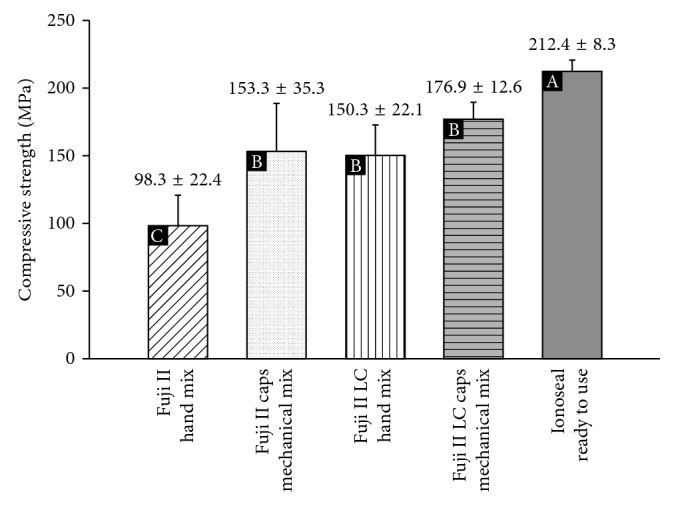
Compressive strength and standard deviation (MPa) of materials tested.

The compressive strength results (MPa) of the tested materials are shown in Figure 1.

The statistical analysis found differences between the materials (*p* < 0.001). Ionoseal presented superior values to the resin-modified GICs (independent of the mixing method) and conventional encapsulated GIC. These materials had similar and higher values of compressive strength than manually manipulated conventional GIC.

**Figure 2:**
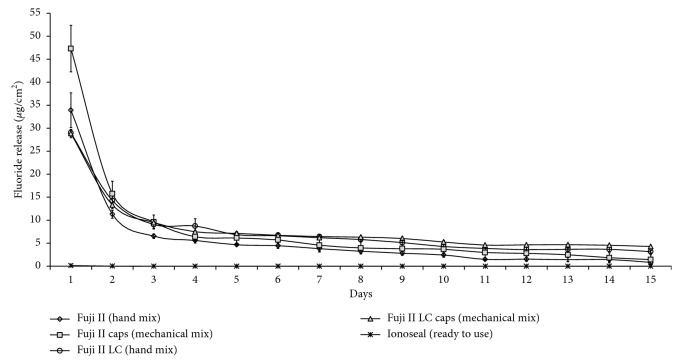
Pattern of fluoride ions release day by day after 15 days.

In the fluoride release analysis, two-way ANOVA revealed significant interactions between material and time (*p* < 0.001). The fluoride release patterns of the GICs tested over a period of 15 days are shown in Table 2. With the exception of Ionoseal, all GICs showed a high release in the first 24 hours and this ions release decreased over time (Figure 2).

In the 24-hour period, Fuji II Capsule released statistically more fluoride when compared to other materials (*p* < 0.05), followed by Fuji II and resin-modified ionomers, regardless of the mixing method. The ready-to-use material (Ionoseal) presented a near-zero release on the first day, and in the other periods of evaluation, no ionic release values were found with this material (Table 2).

**Figure 3:**
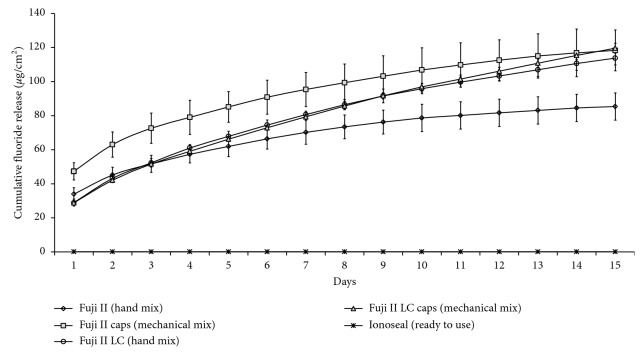
Pattern of cumulative fluoride release after 15 days.

For the second day of evaluation, there was a sudden drop in the fluoride ion release rates for all materials tested, with the Fuji II Capsule showing the highest values (*p* < 0.05) (Figure 2). On the fifth and tenth days of the evaluation, higher values of ion release were observed for Fuji II Capsule, Fuji II LC, and Fuji II LC Capsule (*p* < 0.05). At the fifteenth day of evaluation, the ion release values of the conventional GICs were lower than those of the resin-modified ionomers (*p* < 0.05). At the end of the 15th day, the two resin-modified GICs had cumulative fluoride ion release close to the conventional encapsulated GIC and hand mix ionomer presented lower values. Ready-to-use ionomer cement presented values close to zero (Figure 3).

## 4. Discussion

The mechanical properties of glass ionomers can be influenced by several factors [14, 21–23]. The first hypothesis for this study was that different mixing methods (hand mix, mechanical mix, and ready to use) of the GIC had no influence in the compressive strength of the materials. This hypothesis was rejected.

The ready-to-use material (Ionoseal) presented the highest values of compressive strength when compared to the other materials tested. One of the reasons is that the crosslinked polymer matrices in compomers and composites (typically copolymers of Bis-GMA, UEDMA, and TEGMA) generally have higher strength and toughness than the gel network formed by acid-base reaction in glass ionomers [24]. We found that the difference may also be in the percentage of filler particles, but the particle used in all materials is the same, fluoroalminumsilicate. However, the manufacturer does not give the exact amount of this particle in the composition of the materials tested.

Among the materials that were mixed with different methods, the conventional hand-mixed GIC had the lowest values of compressive strength. The conventional encapsulated GIC (mechanical mix) presented values of compressive strength similar to the resin-modified GIC, independent of the mixing method. For Fleming and Zala [25], the hand mixing technique employed in manipulating the glass ionomer restorative cement maybe results in an even distribution of unreacted glass filler particles in the plastic mass. However, if insufficient force was applied to the cement mass during spatulation, agglomerates form in the plastic cement mass rather than producing an even distribution of powder particles. These powder agglomerates can manifest themselves as crack initiation sites when the material was stressed under load [26]. The commercialization of GICs in the form of prefabricated capsules has emerged to eliminate such interferences from the operator, such as incorrect proportioning of the powder and liquid and the poor manipulation [27].

On the other hand, several researchers have reported that the mechanical properties of encapsulated materials were inferior to those of equivalent hand-mixed materials [12, 25, 27–29]. It is recognized that such mixing methods may result in the incorporation of air porosity in the cement, leading to weakening [15, 27].

The vibratory action of the conventional mechanical mixing machines has been reported to incorporate increased porosity into some encapsulated GIC restoratives [25, 27]. Another mechanical mixing utilizing a combination of rotational and centrifugal action had reduced porosity compared with the conventional vibratory action [15, 25, 27, 30]. The present study used an amalgamator for mixing encapsulated products, which violently shakes the capsule back and forth for 5–15 s and favors the appearance of bubbles, but this was not evident in the results.

However, there are other factors that influence the appearance of bubbles in materials, such as powder/liquid mixing ratio, initial viscosity of the cement mix, capsule design, exit diameter of the nozzle, and even the placement of the material in the matrix can produce bubbles. The method of manipulation did not influence the compressive strength of the resin-modified GICs. We believe that photoactivation is the major responsibility for determining the behavior of the mechanical properties of the material, so the mixing method of the modified ionomers is in the background.

In the same way, the mixing method of the GICs had influence on the release of fluoride ions. Thus, the second hypothesis was rejected. Initially, the acid attacks the glass filler particles. The calcium and aluminum ions are released, forming a salt matrix from the polyacrylate chains within the cement [31]. Fluoride ions are released during the acid attack upon the glass filler and remain within the forming matrix even though they take no further part in the setting reaction [32].

The reason for the high fluoride release of Fuji II Capsule can be explained by the mechanical grinding of the material, which would increase the attack of the polyacrylic acid on the particles of Ca-Al-F-silicate glass and dissolution of these particles, resulting in a greater release of fluoride. The same effect of the mechanical manipulation in the fluoride release was not observed in the modified GIC: the material did not present statistical difference, regardless of the form of manipulation.

All GICs showed a ion release pattern with a high concentration of fluoride in the first 24 hours (“burst effect”), followed by a rapid decline after the second day, with a tendency to stabilize from the second week [23, 33]. An initial high ion release from glass ionomers over the first 24 hours is likely due to the burst of fluoride released from the glass particles when reacting with the polyalkenoate acid during the setting reaction [34]. Another explanation for this is the “cleaning effect” caused by water on the surface of the material with a posterior release of fluoride, which is controlled by the diffusion of the water through the micropores and the cement mass itself [33].

The studies comparing conventional and resin-modified ionomers for fluoride release are controversial: ranging from similar [21], inferior [22] to superior results [23] for the resin-modified cements. In this study, the conventional cements had a higher release in the first 48 hours, with the opposite occurring from the third day, when the resin-modified ones presented a higher release, with constant pattern until the fifteenth day.

Based on the premise that the magnitude and duration of the inhibitory effects of fluoride depends mainly on the concentration and time in which it is retained in the oral cavity, a fluoride release that remains for a longer time is desired to contribute to the longevity of the restorations [35]. This behavior was observed in the resin-modified ionomers used in this study, where it is possible to observe a constant fluoride release rate, besides the high results of resistance to compression.

On the other hand, the release of fluoride ions by the Ionoseal was almost nonexistent on the first day, and from the second day, no release occurred. This may occur due to the silanization of the particles of fluoroalminumsilicate glass. The silane-coupling agent, a bonding agent between filler particles and resin matrix polymer, has been widely used to improve the mechanical properties of the resin composite. Bisphenol-A-glycidyl methacrylate/triethylene-glycol-dimethacrylate (Bis-GMA/TEGDMA) resin containing silanized NaF particles released a small amount of fluoride for a long duration while the resin containing nonsilanized NaF particles released a large amount of fluoride [36, 37]. Some silanes are highly hydrophobic, and this would prevent the water from wetting the surface of the particles and preventing the release of fluoride ions [38, 39].

## 5. Conclusion

Based on the results obtained in this study, the different mixing methods of glass ionomer cements has an influence on its compressive strength and fluoride release.

## Figures and Tables

**Table 1:** Composition of the materials tested in the study.

Material	Fabricante	Composition
Fuji II capsule	GC corp., Tokyo, Japan	*Powder*: calcium fluoroaluminosilicate polyacrylic acid powder, iron oxide *Liquid*: polyacrylic acid (aqueous solution), tartaric acid, water
Fuji II LC capsule	GC corp., Tokyo, Japan	*Liquid*: PAA, HEMA, proprietary ingredient, 2,2,4-trimethyl hexamethylene dicarbonate, TEGDMA *Powder*: (fluoro) alumino silicate glass
Ionoseal	Voco, GmbH, Cuxhaven, Germany	Fluoroalminumsilicate glass, bis-GMA, HEMA, TEDMA, 1,6-hexanediylbismethacrylate

PAA: polyacrylic acid; HEMA: 2-hydroxyethyl methacrylate; TEGDMA: triethylene-glycol-dimethacrylate; bis-GMA: bisphenol A-glycidyl methacrylate; TEDMA: triethylene glycol dimethacrylate.

**Table 2:** Mean and standard deviation of the release of fluoride ions from the materials tested in the evaluation periods of 1, 2, 5, 10, and 15 days.

Materials	Mean and standard deviations of fluoride release (*μ*g/cm^2^)					Cumulative fluoride
	Day 1	Day 2	Day 5	Day 10	Day 15	
Fuji II	33.9 ± 3.7Ab	11.2 ± 0.8Bc	4.6 ± 0.2Cb	2.4 ± 0.5Db	0.8 ± 0.4Db	85.4 ± 8.6
Fuji II Caps	47.3 ± 5.0Aa	15.7 ± 2.7Ba	6.1 ± 1.3Cab	3.6 ± 1.2Dab	1.4 ± 1.0Eb	118.3 ± 11.9
Fuji II LC	28.9 ± 0.7Ac	14.2 ± 1.7Bab	6.8 ± 0.2Ca	4.2 ± 0.4Dab	3.1 ± 0.2Da	113.8 ± 3.9
Fuji II LC Caps	28.8 ± 0.9Ac	13.3 ± 0.1Bb	7.1 ± 0.3Ca	5.2 ± 0.3Da	4.2 ± 0.09Da	119.7 ± 2.0
Ionoseal	0.1 ± 0.1Ad	0 ± 0Ad	0 ± 0Ad	0 ± 0Ac	0 ± 0Ac	0.1 ± 0.1

Different capital letters mean statistical significance within line (*p* < 0.05). Different lowercase letters mean statistical significance within columns (*p* < 0.05).

## Data Availability

The data used to support the findings of this study are available from the corresponding author upon request.
